# The Role of Salicylic Acid in Activating Plant Stress Responses—Results of the Past Decade and Future Perspectives

**DOI:** 10.3390/ijms26094447

**Published:** 2025-05-07

**Authors:** Kincső Decsi, Mostafa Ahmed, Donia Abdul-Hamid, Zoltán Tóth

**Affiliations:** 1 Institute of Agronomy, Georgikon Campus, Hungarian University of Agriculture and Life Sciences, 8360 Keszthely, Hungary; toth.zoltan@uni-mate.hu; 2 Festetics Doctoral School, Institute of Agronomy, Georgikon Campus, Hungarian University of Agriculture and Life Sciences, 8360 Keszthely, Hungary; 3 Department of Agricultural Biochemistry, Faculty of Agriculture, Cairo University, Giza 12613, Egypt; 4 Heavy Metals Department, Central Laboratory for the Analysis of Pesticides and Heavy Metals in Food (QCAP), Dokki, Cairo 12311, Egypt; donia.atalah11@gmail.com

**Keywords:** salicylic acid, abiotic and biotic stress, oxidative stress, priming, nanoparticles, combined application possibilities

## Abstract

Salicylic acid (SA) is one of the most commonly used natural plant protection compounds, considered one of the most effective in mitigating the damage caused by abiotic and biotic stressors. The current review article summarizes the most significant achievements in stress management over the past ten years. We also provide insights into new perspectives on the use of salicylic acid. The article summarizes the role of SA in signaling, its effects on biotic, abiotic and oxidative stress, evaluates the possibilities of its use in combination with other active compounds, and presents the promising application opportunities offered by new techniques that may become available in the coming decades.

## 1. Introduction

Nowadays, the damage caused by global climate change and environmental pollution is causing significant problems, and efforts have been launched worldwide to mitigate them. Plants have many weapons to mitigate these damages. In response to various abiotic and biotic stressors, metabolic changes are induced by information received through signaling pathways. Metabolic changes are often associated with the production of protective compounds. One of the effective protective compounds produced by plants is salicylic acid (SA). In the past decade, researchers have placed increasing emphasis on investigating the effects of the plant hormone salicylic acid, both on signaling pathways and various abiotic and biotic stressors [1,2,3,4].

Recent research has also highlighted that salicylic acid can be activated by stress and a potential growth regulator [5] and immunostimulant plant defense activator [6]. It also plays a key role in some sub-processes of plant morphogenesis [7,8]. Kavulych et al. (2023) reviewed the possibilities of the exogenous applications of salicylic acid in agriculture and plant biotechnology [9]. A summary of knowledge on horticultural crops can be found in the study by Chen et al. (2023) [10], while the beneficial effects of salicylic acid on field crops can be read about for various plant species in the work of Shahrajabian and Sun (2024) [11].

The two main pathways for salicylic acid biosynthesis in plants are phenylalanine ammonia-lyase and the isochorismate pathway [12]. The phytohormone is synthesized from chorismate or phenylalanine [13]. Peng et al. (2021)’s study provides information on the functioning of salicylic acid biosynthesis pathways [14]. In addition to these, Gondor et al. (2016) found that in addition to the main biochemical pathways, the flavonoid metabolic pathway may also be part of the physiological mechanisms triggered by salicylic acid [15].

In our comprehensive review, we report in detail on the new scientific findings of the past decade regarding the effects of salicylic acid. We cover novelties in signal transduction mechanisms, research results related to various abiotic and biotic stressors, the possibilities of reducing oxidative stress, and the application possibilities of salicylic acid in combination with other compounds. Finally, we provide insight into new trends, touching on the combined effects of salicylic acid and nanoparticles, and the application possibilities of salicylic acid as a priming compound.

## 2. Discussion

### 2.1. The Results of the Past Decade

#### 2.1.1. Salicylic Acid and Signal Transduction

In the past ten years, several authors have studied and summarized the knowledge regarding the salicylic acid signaling pathway in review publications [16,17,18]. Jia et al. (2023) provide insights into the early evolution and diversification of plant SA signaling and biosynthesis pathways and conclude that salicylic acid plays a vital role as a signaling molecule at all levels of plant evolution [19].

Increased levels of SA are observed in many stress processes. In their review article, Chen et al. (2020) summarize the role of transcription factors and epigenetic regulatory molecules in the signaling of SA-mediated immune responses [20]. Several essential regulators regulate SA accumulation. Defense-related signaling genes affecting SA production have been discovered in succession, such as Enhanced Disease Susceptibility 1 (EDS1), Phytoalexin Deficient 4 (PAD4), Senescence-Associated Gene 101 (SAG101), and Non-Race-Specific Disease Resistance 1 (NDR1) [21,22,23]. In addition, the role of several transcription factors as modulators of SA signaling has been elucidated, such as SARD1 and CBP60g [24] WRKY28 [25], and a transcription complex containing TCP proteins [26].

Salicylic acid activates protein kinases, which can phosphorylate and activate transcription factors involved in stress responses [27]. Some proteins that bind to SA play essential roles in plant immunity. However, only the members of the “non-expressor of PR-protein” family, NPR1, NPR3, and NPR4 can be considered true SA receptors [28,29,30]. Janda and Ruelland (2015) discovered that a transcription factor, named “non-expressor of PR-protein 1” (NPR1), plays a role in the production of SA and regulation of salicylic acid levels [31]. When salicylic acid amounts change, it sets off a chain of events that make different stress response proteins work together to control signals. The genes encoding these proteins are activated in response to stress. Hernández et al. (2017) found that the signaling molecule affects the antioxidant defense system by interfering with the redox system through the NPR1 redox-regulated protein [32]. As a result, transcription factors are activated and the transcription of defense genes is initiated.

Lai et al. (2018) showed that the transcription factor NPR1 modulates essential protective functions during stress in the endoplasmic reticulum (ER) [33]. If this function did not exist, the ER would not be able to perform its function under stress conditions, leading to the death of the eukaryotic cell. Stress leads to the accumulation of misfolded proteins in the ER, which prompts plants to increase the degradation of such proteins in response. This response is called the unfolded protein response (UPR) [34]. Meng et al. (2017) discovered that the salicylic acid signaling pathway also plays a vital role in establishing the UPR [35]. Pokotylo et al. (2019) revealed that NPR1 is a key regulator of the salicylic acid biochemical signal transduction pathway. Still, they also observed several SA-induced responses that do not require the mediation role of the NPR1 molecule [36]. In contrast to NPR1, which positively regulates SA-mediated plant immunity, NPR3 and NPR4 function as negative regulators of plant defense [28,37].

However, SA’s role in signaling processes is much more diverse, and the diversity of processes it regulates suggests that these processes cannot be activated by a single or a few transcription factors. Several candidate SA signaling protein molecules have been observed, suggesting that SA signaling can be mediated through multiple pathways. To our current knowledge, this is not typical for other plant hormones.

Dempsey and Klessig (2017) showed that SA can bind to several plant stress-inducible proteins, altering their activity [38]. This finding differs from the idea that hormones mediate their functions through one or a few receptors. Several authors [39,40] have also shown that the PBS3 protein plays a role in SA biosynthesis.

Song et al. (2023) elaborate on the role of genes involved in enhancing plant immunity in salicylic acid signaling pathways [41]. Yang et al. (2022) found that metabolic and transcriptional analyses revealed 72 and 5469 differentially expressed metabolites and genes under heat and drought stress, respectively, several of which are genes of the salicylic acid signaling biochemical pathway [42]. The results support the hypothesis that the SA signaling pathway can also be activated by multiple abiotic stresses and may play a role in triggering defense responses.

Ahmad et al. (2019) found that salicylic acid modulates the mitogen-activated protein kinase (MAPK) signaling cascade, thereby indirectly stimulating the expression of genes encoding some antioxidants and secondary metabolites, heat shock proteins (HSPs) and chaperones, as well as sinapyl alcohol dehydrogenase (SAD), cinnamyl alcohol dehydrogenase (CAD), and cytochrome P450 [43]. Liu et al. (2022) described in their work that salicylic acid acts as a regulator through signaling pathways on additional physiological processes, such as the function of abscisic acid (ABA), calcium ions, or even nitric oxide [16]. This seems to confirm that hydroactive stomatal closure, as a stress-induced process, is also not independent of SA signaling. Belt et al. (2017) say that the rise in the activity of the succinate dehydrogenase (SDH) enzyme in mitochondria is closely connected to signaling that depends on salicylic acid. When the plant is stressed, an increase in salicylic acid concentration leads to an increase in SDH enzyme activity and increased hydrogen peroxide (H_2_O_2_) production, which triggers transcriptional responses in the plant [44]. Santisree et al. (2020) reported that the salicylic acid signaling pathway is linked to the jasmonic acid signaling pathway, and their combined action regulates plant resistance to stressors [45].

In addition, SA can induce epigenetic changes, as its accumulation can modify the structure of chromatin. These chromatin modifications then lead to changes at the transcriptional level [46]. Epigenetic changes can also be induced by influencing certain enzymes (e.g., histone deacetylases). Studies have shown that histone deacetylases (HACs) are involved in biotic and abiotic stress responses [46,47,48]. Histone deacetylases, a group of enzymes that remove acetyl groups from histones, cause histones to be more tightly wrapped around DNA, making the DNA less accessible to transcription factors. A 2018 study showed that HACs are essential for SA-mediated immunity and the expression of genes involved in the SA pathway [49].

Another family of enzymes, histone demethylases, removes methyl groups from histone proteins and is also involved in SA-mediated immunity. Dutta et al. (2017) showed that some histone demethylases modulate responses to pathogens [50]. In plants, DNA methylation plays a vital role in silencing transposon elements and endogenous genes and can also influence chromatin structure. López Sánchez et al. (2016) reported that DNA methylation plays a negative role in SA-mediated plant defense by reducing the expression of defense genes [51]. In addition to the above, small RNAs play a key role in the epigenetic regulation of plant immunity [52].

Spoel and Dong (2024) concluded in their summary that the regulatory mechanism of salicylic acid does not occur in a single pathway but involves complex networks that allow for multilevel regulation both locally and throughout the plant organism [53]. The system includes inducible genes, transcription factors, a redox system, certain enzymes, and other proteins that connect several subcellular compartments through their location and function. It can also be said that the salicylic acid molecule controls several post-translational events, which form protein condensates that act as signal transduction nodes. They also mention that salicylic acid interacts with other hormones in signal transduction pathways, thus exerting a combined, complex effect. In addition to the above, it can be considered a relatively novel discovery that photoreceptors that mediate light and its effects interact with transcription factors modulated by the salicylic acid pathway. Thus, the salicylic acid pathway also plays a role in mediating phototransduction processes [54].

#### 2.1.2. Salicylic Acid and Salt Stress

Among abiotic stressors, salt stress has become the most important today, as it is the leading cause of food supply problems for most people worldwide. That is why, in the past decade, many research groups have set the primary goal of curbing the effects of salt stress. In the past decade, several comprehensive works have been prepared on the interactions between salicylic acid and salt stress, among which Jayakannan et al. (2015) focused on the role of salicylic acid in ion transport processes [55] and Bhuvaneshwari (2017) reviewed experiments with salicylic acid to eliminate salt stress in various plant cultures [56]. A review by Hoque et al. (2020) highlighted the role of salicylic acid in signaling cascades [57], which exerted its physiological effects mainly through modulating sodium and potassium transporters. Sharma et al. (2023) detailed the physiological and biochemical processes by which salicylic acid eliminates salt stress [58], while Yang et al. (2023) focused on the molecular responses induced by salt stress [27]. In addition to the review studies, several research groups have set up field experiments to reduce the effects of salt stress by applying exogenous SA.

Mimouni et al. (2016) investigated the effects of salicylic acid on tomato plants under salt stress conditions [59]. They found that SA restored normal photosynthetic rates and desirable photosynthetically active pigment levels in the plants. Jini and Joseph (2017) investigated the effects of SA on eliminating salt stress damage on rice plants [60]. They found that externally applied SA increased the exogenous SA level of the plant. It reduced the uptake of Na^+^ and Cl^−^ ions and generally alleviated salt stress symptoms. Farhangi-Abriz and Ghassemi-Golezani (2018) studied salt-stressed soybeans and found that exogenously applied one mM SA increased total biomass and yield [61]. It also increased leaf chlorophyll and water content, along with calcium, potassium, and glycine-betaine content. Tahjib-Ul-Arif et al. (2018) conducted experiments on maize plants. They found that SA administered during salt stress positively affected photosynthetic parameters [62], antioxidant enzyme activity, and reduced membrane damage. Naeem et al. (2020) investigated the beneficial effects of salicylic acid on tomato plants under salt stress [63]. They found that SA applied at a concentration of 0.5 mM significantly alleviated the adverse effects of salt stress on fruit quality and quantity parameters.

From the above seemingly completely different examples, it is clear that SA does not act through a single signaling pathway but is involved in several defense mechanisms and modulates several biochemical pathways. Its mechanism of action is broad-spectrum and can exert its plant protective role in several places. Numerous other publications document additional beneficial effects of salicylic acid treatments and the mechanisms of action mentioned above. Due to the scope of the literature sources available in the past decade, we summarize additional studies published in tabular form (Table 1).

**Figure 1:**
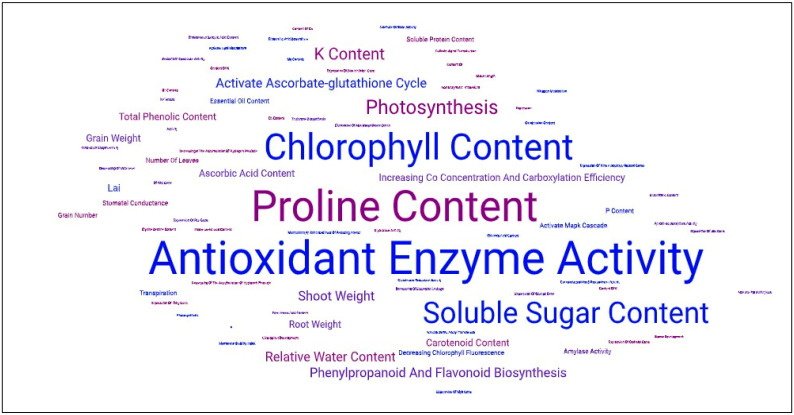
The biochemical processes most responsive to SA treatments under salt stress conditions. The font size is proportional to the hit rate of the literature references for biochemical processes affected by SA.

All of the above references consistently present experiments in which SA application benefits certain plant physiological parameters and biochemical processes. However, the research results above and in the tables suggest that the main areas of action of SA in salt stress protection may be antioxidant enzyme activity, proline content, chlorophyll content, soluble sugar content, and photosynthetic processes (Figure 1).

By activating the antioxidant enzyme system, SA promotes the elimination of ROS, whereby the enzymes bind the ROS members instead of the activated electrons resulting from the damage of the primary stressor [125]. As a result, peroxidation of the lipid layers of the membranes is prevented, so the malondialdehyde (MDA) level also remains low. Low MDA levels do not further damage cells exposed to stress [126].

The increase in proline levels can be understood as a primary stress response. It can repair damaged cells, as it is easily degraded and is a source of carbon and nitrogen in the assimilative processes. As an osmolyte, it maintains the shifted concentration conditions of the cells, preventing further water loss and thus maintaining their homeostasis [127]. The relative water content also increases due to this process compared to the water content of stressed but untreated plants [128]. The increased chlorophyll content due to SA may promote an increase in the efficiency of photosynthesis [129].

An increase in soluble sugar content is also characteristic of stressed plants following SA treatment, so they also play a particularly important role as ROS scavengers [130]. The acceleration of the ascorbate–glutathione cycle following SA treatment may be of particular importance, as this is one of the most efficient biochemical pathways of detoxification processes in plants [131].

Furthermore, the positive effect of SA on the secondary metabolic pathways of plants is also important, such as phenylpropanoid and flavonoid biosynthesis, as these pathways produce secondary metabolites that also actively participate in antioxidant reactions [132]. The increase in the levels of carotenoids [133] and ascorbic acid [134] also support the strengthening of antioxidant defense mechanisms.

#### 2.1.3. Salicylic Acid and Drought Stress

Drought is the second-most problematic abiotic stressor worldwide. The fact that several research groups have been working on mitigating its harmful effects for the past decade further underscores its importance. Salicylic acid plant conditioning treatment can also be an effective tool to eliminate its harmful effects. Manzoor et al. (2015) and colleagues conducted a study on the exogenous application of salicylic acid [84]. Foliar treatments increased both shoot and root dry matter content and increased the amount of various amino acids, most notably proline content. Since proline acts as an osmolyte, higher relative water content was measured in conditioned plants due to its protective effect.

Latif et al. (2016) investigated the effects of SA on maize during drought stress [135]. The beneficial effects were attributed to an increase in the soluble and bound phenolic content of the treated plants. In addition, the total soluble protein content and relative water content of the plants also increased. Noreen et al. (2017) conducted an experiment on wheat and found that the biomass reduction under stress was alleviated by the application of SA [119]. Total soluble protein content and total amino acid content also increased. Water management indices of the treated plants improved, and the 1000-seed weight also increased.

Shan et al. (2024) investigated the effects of salicylic acid against drought stress [107]. The researchers conducted leaf proteome profiling studies during the vegetative and generative phases. They found that the treated plants had more proteins during photosynthesis and ATP synthesis. Carbon (C) metabolic proteins involved in osmoregulation and C-partitioning alleviated the damage caused by drought. In addition, the degradation mechanisms of stress-damaged proteins also showed more active functioning. All of these results show that applying salicylic acid from the outside affects the way proteins work in many ways and plays a big part in protecting cells from drought stress. In plants, SA-binding protein 2 (SABP2) with methyl salicylate (MeSA) esterase activity catalyzes the conversion of MeSA to SA. Li et al. (2019) cloned a salicylic acid binding protein from Lycium chinense (LcSABP) [136]. The overexpression of LcSABP enhanced drought tolerance in transgenic tobacco plants. The results indicated that increased levels of LcSABP transcripts and endogenous SA content play a role in enhanced drought tolerance.

La et al. (2019) found that treating cells with SA decreased ROS activity and increased the expression of genes that make proline (P5CS1, P5CS2, and P5CR) [137]. NPR-1 and PR-1 genes were also up-expressed, confirming their role in signal transduction. Moreover, the SA treatments induced the GRX-9 gene, known for its association with redox potential changes. Estaji and Nikham (2020) sprayed milk thistle plants with salicylic acid and found that the osmotic balance of the treated plants was almost completely restored compared to untreated and drought-stressed plants [138]. In addition, the composition and quality of the plants’ seed oil also improved.

Khalvandi et al. (2021) subjected winter wheat under drought stress to SA treatment [139]. Salicylic acid treatments effectively alleviated the negative effects of drought stress by enhancing the activity of antioxidant enzymes, preserving membrane permeability, improving photosynthetic performance, and inducing the production of stress proteins. Aires et al. (2022) investigated the beneficial effects of SA in tomato [140]. They found that the treatments reduced the number of aborted flowers, increased CO_2_-assimilation, and also increased the efficiency of carboxylation.

Silva et al. (2023) treated watermelon seedlings with salicylic acid under drought stress conditions [141]. They found that the treatment reduced electrolyte leakage and positively affected the concentration of organic solutes. Their studies revealed a new property of externally applied salicylic acid: its role as a metabolic support. Shan et al. (2024) studied rice plants under drought stress [107]. They found that the expression levels of some stress-induced genes (OsDREB2A and OsSAPK8) increased in SA-treated plants. They concluded that the antioxidant capacity of the plants increased as a result of the treatments, counteracting the harmful effects of stress. It follows from the above that the exogenous application of the salicylic acid molecule can counteract the harmful effects of drought stress in many ways and through multiple points of attack. Table 2 below lists additional scientific literature written in the last decade focusing on the most important areas of action of salicylic acid vs. drought stress.

**Figure 2:**
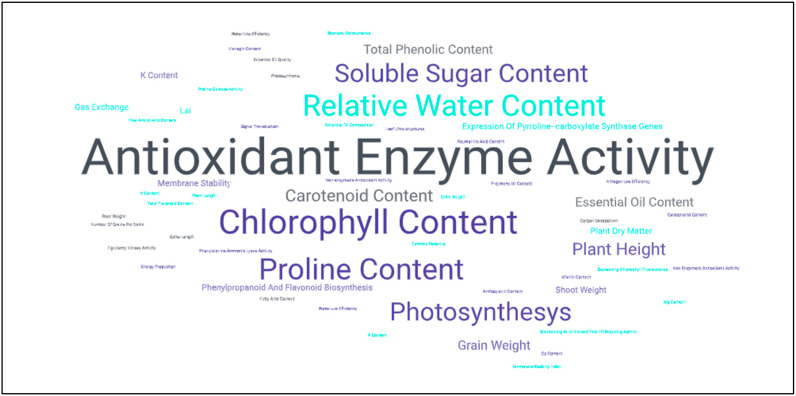
The biochemical processes most responsive to SA treatments under drought stress conditions. The font size is proportional to the hit rate of the literature references for biochemical processes affected by SA.

Essentially, it can also be stated that the main areas of action of external application of SA in drought stress—similar to eliminating the effects of salt stress—may be antioxidant enzyme activity, chlorophyll content, proline content, and relative water content (Figure 2). The biochemical pathways modulated in drought stress following SA treatments are completely identical to the metabolic pathways induced in salt stress.

#### 2.1.4. Salicylic Acid and Heavy Metal Stress

About 11% of the Earth’s soils are considered fertile without restrictions. The remaining 89% are soils exposed to some damaging effect. About 23% of this, or 89%, are chemically damaged soils. This also includes soils containing heavy metal contamination. However, a high heavy metal content in the soil does not mean it is toxic to plants, since only mineral elements in dissolved and soluble (absorbable) forms affect plants. The following mechanisms characterize the mechanism of heavy metal toxicity in plants:

Damage to the plasma membrane, changes in the dynamics of transport processes (decrease in ATPase activity, increase in K+ efflux), development of oxidative stress (Fenton and Haber–Weiss reactions), increased lipid peroxidation and malondialdehyde synthesis, appearance of free metal ions in the cytoplasm, and cell cycle and cell elongation disorders. We can use salicylic acid as a bioactive agent to combat these harmful effects. In the past decade, numerous studies have focused on the elimination of various heavy metal stresses with salicylic acid treatments.

Liu et al. (2016) focused on the effects of cadmium stress and the potential for its elimination by salicylic acid in their review [177]. Guo et al. (2019) reviewed the effects of salicylic acid treatments against cadmium stress [178]. They concluded that modifying reactive oxygen species levels may primarily influence SA signaling mechanisms in resolving cadmium stress. Sharma et al. (2020) summarized the responses of plants to heavy metal stress after salicylic acid treatments [179]. The authors concluded that salicylic acid and other plant hormones promote the activation of the antioxidant defense system and help reduce heavy metal loads. SA application increases plant tolerance to heavy metal stress by modulating the levels of several metabolites, including components of the antioxidant defense cascade, secondary metabolites, osmolytes, and metal chelation mechanisms [180].

Singh et al. (2015) investigated eliminating arsenic loads in rice. SA helped inhibit the translocation of the heavy metal in the plant and mitigated the effects of secondary oxidative stress [181]. Mei et al. (2015) investigated the effects of copper overload stress on cotton plants and its elimination potential by salicylic acid [182]. They found that although copper uptake was not inhibited, its toxic effects were significantly reduced due to the antioxidant responses activated by the treatments. Shakirova et al. (2016) investigated the potential for reducing the toxic effects of cadmium in wheat [183]—salicylic acid treatment reduced electrolyte leakage and malondialdehyde accumulation. In addition, the enzyme phenylalanine ammonia-lyase was activated, promoting lignin deposition in plant cell walls. The effects of the treatments were accompanied by dehydrin accumulation, which was beneficial.

Saidi et al. (2017) investigated the effects of arsenic loading on sunflowers. A high level of oxidative stress was observed, which was reduced by the application of SA [184]. The treatments increased the activities of catalase, ascorbate peroxidase, and glutathione peroxidase enzymes. Agnihotri et al. (2018) studied *Brassica juncea* under lead stress [185]. They found that oxidative stress induced by lead exposure damages DNA structure. However, SA treatments enhance the function of the ascorbate–glutathione cycle, which has a powerful detoxifying effect.

Zaid et al. (2019) exposed mustard plants to nickel stress. This stressor increased the level of hydrogen peroxide in the plants, which had a detrimental effect on the structure of the membranes [186]. This led to lipid peroxidation, which caused electrolyte leakage. Methylglyoxal and malondialdehyde levels also increased. However, exogenous foliar application of salicylic acid activated the ascorbate–glutathione (AsA-GSH) cycle and the glyoxalase system, thus reducing the degree of stress.

El Dakak et al. (2020) also concluded that salicylic acid treatments used to counteract cadmium loading help maintain the redox balance of plants and also reduce ROS loading [187]. Gupta and Seth (2021) investigated the effects of chromium pollution on tomatoes. In addition to the previously listed adverse effects, they observed a decrease in the amount of photosynthetically active pigments and total gas exchange activity [188]. However, 0.5 mM SA treatment restored normal vital functions. This was because the ascorbate–glutathione cycle was revved up, helping eliminate the harmful ROS activity.

Kaur et al. (2022) investigated cadmium loading in mustard [13]. They found that cadmium imbalanced macronutrient and Ca uptake and oxidative stress. Moreover, they observed reduced photosynthetic PSII activity and the dark phase’s characteristic gas exchange efficiency. SA reversed the detrimental effects and normalized homeostasis. In Table 3 below, we present in tabular form the other most important publications on this topic of the past decade.

**Figure 3:**
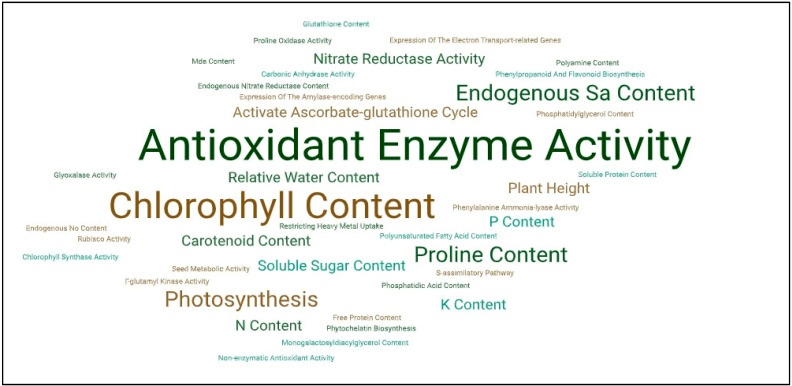
The biochemical processes most responsive to SA treatments under heavy metal stress conditions (The font size is proportional to the hit rate of the literature references for the biochemical processes affected by SA).

Overall, it can be concluded that in addition to activating the antioxidant system, chlorophyll content, endogenous SA content, proline content, and photosynthesis modulation can also be observed (Figure 3). Similarly to the abiotic stressors discussed above, the same biochemical pathways were affected by SA treatment. In addition, the endogenous SA level increased following exogenous treatments, which supports the plant organism with additional protective effects [75].

#### 2.1.5. Salicylic Acid and Heat Stress

Higher than optimal temperatures reduce the yield of cultivated plants. This is causing increasing problems in many countries around the world. As a result of global climate change, it can be assumed that the frequency of hot periods, also affected by atmospheric drought, will increase; therefore, studies related to increasing heat tolerance will also become increasingly important in the future. As a bioactive agent, salicylic acid has inspired several research groups to explore its potential beneficial effects on heat tolerance. Nazar et al. (2017) published a study on plant responses to heat stress and their support with salicylic acid [210]. Iqbal et al. (2019) devoted an entire chapter to summarizing the knowledge about salicylic acid as a compound inducing plant defense responses and examining its interactions with other plant hormones [211]. Rai et al. 2020 summarized the molecular genetic correlates of the role of salicylic acid in heat tolerance [212]. They talked about the transcriptional level of salicylic acid-induced signaling processes that make plants more tolerant of heat. They also talk about its role in creating stress memory. In their review, Sangwan et al. (2022) summarized the knowledge on the external application of salicylic acid during heat stress, with particular attention to its role in inducing the production of heat shock proteins, osmolytes, secondary protective metabolites, and antioxidants [213]. A study by Janaagal et al. (2024) was also published on a similar topic [214].

Khanna et al. (2016) successfully applied SA supplementation in heat-sensitive maize hybrids [215]. The treatments helped the Halliwell-Asada pathway, which raised the activities of the enzymes ascorbate peroxidase and glutathione reductase. One of the most important biochemical pathways with antioxidant effects was turned on. Cingoz and Gurel (2016) investigated whether salicylic acid treatment affected the cardenolide content of Digitalis trojana plants under heat stress [216]. Cardenolide is a glycoside used to treat heart failure. After being treated with salicylic acid, the plants made a lot more cardenolide and had higher amounts of proline, phenol, and flavonoids. The production of all these metabolites demonstrates the inductive effect of salicylic acid on protective responses against stress.

Martel and Qaderi (2016) treated pea plants exposed to heat stress with salicylic acid [217]. The treatment had a beneficial effect on some growth characteristics. It significantly increased net CO_2_ assimilation and positively affected chlorophyll composition and content, but did not affect chlorophyll fluorescence and the production of additional secondary metabolites or protective compounds. Zhang et al. (2017) examined heat-stressed rice plants with SA treatment and evaluated the changes in spikelet development [218]. The treatments can eliminate the extensive spikelet degeneration that high temperature causes. Salicylic acid had a beneficial effect on all spikelet and yield parameters. In addition, some antioxidant enzymes (catalase, ascorbate peroxidase, peroxidase, and superoxide dismutase), plant hormones, soluble sugars, and proline content increased in the treated plants. Similar conclusions were reached by Feng et al. (2018) after treating heat-stressed rice plants with salicylic acid [219]. SA stopped reactive oxygen species (ROS) from building up in the anthers, which stopped oxidative stress-related programmed cell death (PCD) in tapetum cells. Genes related to tapetum development were also involved in this process. These genes include microsporeless (MIL2), eternal tapetum 1 (EAT1), and defective tapetum meiosis 1 (DTM1). Thus, SA seems to indirectly benefit pollen cell survival under heat stress and fertilization success.

Afzal et al. (2020) conducted experiments with wheat under heat stress [220]. Plants treated with salicylic acid showed a measurable increase in catalase and peroxidase activity and proline content, which is consistent with previous research groups’ results. Shah Jalan et al. (2019) treated tomato plants exposed to heat stress with salicylic acid and found that the treatments had a beneficial effect on the antioxidant system, photosynthesis, and water uptake processes [221]. SA prevented membrane damage, thereby reducing electrolyte leakage. It increased the amount of chlorophyll and carotenoids, PSII improved efficiency, and improved overall gas exchange. In addition, water management became more stable, among other things, due to an increase in the level of the osmoprotectant proline. Zhang et al. (2020) investigated the application of salicylic acid to ornamental pepper plants during heat stress [222]. They found that the plants’ heat tolerance increased due to the treatments, which was due to the activation of the antioxidant system and an increase in photosynthetic capacity. Similar results were obtained by Wassie et al. (2020) in alfalfa [223].

Overall, it can be stated that, similarly to the previously discussed abiotic stressors, SA mainly supports the antioxidant capacity of plants and the functioning of metabolic pathways involved in detoxification. In addition, it promotes the production of secondary protective compounds, ensuring the elimination of stress-related damage. Cell membranes remain intact, and electrolyte leakage is reduced. SA treatment triggers the production of insoluble sugars and increases the proline content. This also improves the water balance of the plant. The photosynthesis of treated plants also works more efficiently than that of untreated plants.

#### 2.1.6. Salicylic Acid and Cold Stress

As a result of global climate change, our world’s weather has become more extreme, so in many cases, the unexpected onset of cold periods causes stress. In this context, several research groups have also investigated the possible beneficial effects of salicylic acid. Saleem et al. (2021) in their report summarized that salicylic acid exerts its effects against cold stress through multiple signaling pathways, such as the ABA-dependent or -independent pathway, Ca^2+^ signaling pathway, mitogen-activated protein kinase pathway, reactive nitrogen and oxygen species pathways, etc. [224]. The signals flowing through the mentioned pathways produce antifreeze proteins, dehydrins, osmolytes, and antioxidants, helping the plant in its defense.

In the past decade, Orabi et al. (2015) subjected tomato plants to salicylic acid treatment under cold stress [225]. The treatments made more abscisic acid, indoleacetic acid, and gibberellic acid. They also made more of the enzymes peroxidase and polyphenol oxidase. This resulted in significant improvements in the quantitative and content parameters of the crop compared to cold-stressed but untreated plants. However, they also observed reduced catalase enzyme activity in the treated plants. Similar results were obtained by Mutlu et al. (2016) in barley, with the difference that, in addition to the antioxidant enzymes mentioned above, an increase in the level of superoxide dismutase was also detected [226].

Huang et al. (2016) treated *Dendrobium officinale* plants with salicylic acid, which alleviated the adverse effects of cold stress [227]. SA significantly increased superoxide dismutase activity and decreased ascorbate peroxidase and catalase activities. The plants’ net photosynthetic rate and maximum photochemical efficiency were significantly increased compared to the control. Keshavarz et al. (2016) investigated the effects of salicylic acid treatment on cold stress in rapeseed [228]. They found that the levels of peroxidase and superoxide dismutase enzymes increased, but catalase enzyme activity decreased. In addition, the total protein content and proline levels of treated plants also increased.

Wang et al. (2021) investigated the damaging effects of cold stress in wheat and the potential mitigating effects of salicylic acid treatments [229]. They showed that the treatments turned on the plant’s entire antioxidant defense system, boosted PSII activity, and encouraged the production of osmoprotectants, which made the plants more resistant. However, they failed to find convincing evidence for the expression of defense responses at the gene level. Wang et al. (2023) investigated the responses of alfalfa to cold stress after salicylic acid treatment using transcriptome analysis [230]. They found that the treatments induced the phenylalanine ammonia-lyase metabolic pathway. They also showed an increase in the expression of some genes of the SA signaling pathway (MPK3, MPK9, WRKY22, TGA1). These genes can be indirectly linked to the production of antioxidant enzymes peroxidase (POD), ascorbate peroxidase (APX), and glutathione-S-transferase (GST) through the upregulation of the ascorbate–glutathione cycle. To summarize the experience, similar to the previous stressors, an increase in antioxidant capacity, proline production, and photosynthetic activity was observed following SA treatments.

#### 2.1.7. Salicylic Acid and Nutrient Stress

Plants can absorb different concentrations of nutrients in soils, and their nutrient requirements differ significantly between plants. The nutrient uptake and supply of plants is not always optimal. Unfortunately, neither a deficiency nor a surplus is good for plant organisms, and a surplus, once it enters the food chain, can be extremely harmful to subsequent users. Both a deficiency and a surplus are abiotic stress effects, and some researchers have also tried salicylic acid treatments to reduce the harmful effects.

Per et al. (2017) summarized the interactions between salicylic acid and nutrient metabolism in their book chapter [231]. They also discuss the stress effects caused by the lack or excess of micro- and macronutrients and the possibilities of eliminating them using salicylic acid. Namdjoyan et al. (2017) investigated the effects of salicylic acid in safflower under zinc stress [232]. The treatments significantly increased root and shoot length compared to untreated, zinc-stressed plants. In addition, ascorbate and glutathione levels increased in nutrient-stressed plants treated with salicylic acid. The results suggested that salicylic acid had a beneficial effect on the activities of enzymes involved in the ascorbate–glutathione cycle and the glyoxalase system.

Metwally et al. (2018) treated rapeseed plants suffering from boron excess with salicylic acid and observed that the treatments reduced the harmful boron accumulation in the plants and improved water balance parameters [233]. Salicylic acid treatment normalized the activity of ascorbate and dehydroascorbate levels, which increased due to boron toxicity. The glutathione and flavonoid content of the treated plants did not change compared to the control. Similarly, El-Shazoly et al. (2019) examined wheat plants under boron stress [234]. Plants treated by salicylic acid showed boron tolerance, which was not reflected in the limitation of boron uptake but was indicated by increased amino acid, soluble protein, and carbohydrate content.

Moustafa-Farag et al. (2020) treated watermelons suffering from boron toxicity and found that salicylic acid alleviated boron stress-induced chlorosis by increasing chlorophyll and carotenoid content in treated plants [235]. It also stopped the buildup of more boron and helped photosynthesis return to normal. It also turned on superoxide dismutase, peroxidase, and ascorbate peroxidase. Farghaly et al. (2021) treated tomato plants with salicylic acid and found that the increased non-protein thiol (NPT) content due to toxicity was normalized by salicylic acid treatment [236]. The latter compounds are considered antioxidants that act through various mechanisms, e.g., by forming metal chelates and detoxifying the plant organism. These results seem to support the hypothesis that salicylic acid has a stress-relieving effect.

In contrast, Deus et al. (2020) attempted to reduce the symptoms of nitrogen deficiency in rice plants by treating them with salicylic acid, but unfortunately no change was observed [237]. From this, they concluded that salicylic acid does not modulate nitrogen metabolism pathways.

Li et al. (2022) treated grapevines under selenium stress with SA [238]. They observed that the treatments increased superoxide dismutase activity but decreased peroxidase levels and had no effect on catalase. The treatment had a positive effect on the shoot and root biomass of the plants compared to untreated, selenium-stressed plants.

The contradictory results probably stemmed from the fact that symptoms of nutrient excess can be eliminated by salicylic acid, as the excess nutrient is bound. However, the missing nutrient cannot be replaced by SA alone. In this case, the effect of SA had a beneficial effect on detoxifying metabolic processes and was mainly involved in mitigating the toxic effects of excess nutrients.

#### 2.1.8. Salicylic Acid and Alkaline Stress

The properties of bedrock dictate the range of economically viable crops that can grow on it. In the few areas that can be farmed, there are also soils where the properties of the bedrock are made worse by bad nutrient management, which makes the pH change strongly towards alkalinity. In the past decade, some researchers have tried to increase alkali tolerance with salicylic acid treatments. Nie et al. (2018) investigated the effects of abiotic stress induced by alkaline soil on cucumber plants. Alkaline pH caused ion uptake disorders, thereby disrupting plant homeostasis [239]. In addition, harmful oxidative stress occurred, photosynthetic activity decreased, and electrolyte leakage began. Salicylic acid treatments induced the ascorbate–glutathione cycle, reduced lipid peroxidation, and normalized ion metabolism.

Khan et al. (2019) grew tomato plants at pH 9 and found that these plants behaved similarly to cucumber plants in Nie’s experiment under alkaline stress [240]. However, treatment with salicylic acid greatly reduced oxidative stress. At the same time that antioxidant levels rose, the stress hormone abscisic acid levels dropped in the plants. The treatment restored the endogenous Na^+^/K^+^ content and ratio, shifting it towards the K^+^ content. The authors concluded that salicylic acid achieves its beneficial effect against alkaline stress by activating key enzymes of the antioxidant system and modulating endogenous hormone levels.

#### 2.1.9. Salicylic Acid and Biotic Stress

It has long been known that in addition to abiotic stress, the salicylic acid signaling pathway and endogenous salicylic acid also play a role in plant defense against biotic stress. SA is a key component of plant defense, and it accumulates during various plant resistance responses. Salicylic acid itself can also activate the plant immune system for increased resistance to pathogen attack [241]. Qi et al. (2018) investigated the synthesis of salicylic acid through the isochorismate synthase pathway during biotic stress [23]. Similar mechanisms are described in the following review articles by Lefevere et al. (2020) and Rekhter et al. (2019) [39,242]. The transcriptional coactivator NPR1, as a master regulator of salicylic acid signaling, interacts with transcription factors and induces the expression of antimicrobial pathogenesis-related (PR) genes. In this work, strategies used by individual plant pathogens to eliminate the protective role of salicylic acid are detailed. Researchers have long known that SA is essential for innate immunity, but it also plays an important role in the development of systemic acquired resistance (SAR). A study of its role in SAR was published by Klessig (2016) and Mohamed et al. (2020) [8,243]. Maruri-López et al. (2019) reported that SA initially accumulates in locally infected tissues, then spreads throughout the plant and induces SAR in distant, uninfected parts of the plant [244]. Their review discussed the translocation pathways contributing to SAR and their genetic regulation.

Ding and Ding (2020) summarized the latest knowledge on SA biosynthesis and transcriptional regulation of SA biosynthesis, and their role in defense against biotic stressors in their review article [12]. In the meantime, several research groups have begun to focus on finding compounds that promote the synthesis of endogenous salicylic acid—as a protective compound. That was discussed in the study by Urban et al. (2022) [245].

#### 2.1.10. Salicylic Acid and Oxidative Stress

Most noticeable or measurable signs of stress are linked to changes in oxygen metabolism. During this time, there are more homolytic (one electron transfer) processes than heterolytic (two electron transfer) processes, and free radicals are created. Primary stress effects damage cell membranes, releasing electrons from the electron transport chain. When these electrons come into contact with oxygen, they produce reactive oxygen species. The damage equalizes the potential difference on both sides of the damaged membrane, preventing the formation of ATP. Thus, there is no ATP formation, no energy production, and the cell dies.

ROS are made up of free radicals, like superoxide (O_2_^•−^) and hydroxyl radical (^•^OH), which have an extra electron. They are also made up of molecules, like hydrogen peroxide (H_2_O_2_) and singlet oxygen (^1^O_2_), that can turn into free radicals during their reactions. These radicals and molecules are formed from molecular oxygen by a series of reductions, are extremely reactive, and are destructive. They have a detrimental effect on the biosynthesis of nucleic acids, proteins, and lipid components (mainly seeking unsaturated fatty acid molecules of the membrane as reaction partners). They can cause mutations, aging, and apoptosis (cell death) due to severe abiotic stress or infections. They are usually produced abnormally in the plasma membrane during photosynthesis and respiration. ROS are also toxic to plants, which is why detoxification of the body is necessary.

In the past decade, many researchers have focused on ROS neutralization and have tested various plant-based active ingredients. It seems that salicylic acid may be a successful choice in this area as well. Herrera-Vásquez et al. (2015) studied the relationship between salicylic acid and its interacting reactive oxygen species and glutathione (GSH) in stressed plants [246]. They focused on linking SA, ROS, and GSH signals to the transcriptional regulation of defense genes. Arif et al. (2020) reviewed the signaling role of salicylic acid in oxidative post-translational modifications and in eliminating the effects of ROS in their review [247]. In his study, Poór (2020) discussed the role of salicylic acid in mitochondrial ROS metabolism and highlighted the practical implications of using salicylic acid in crop protection [34]. Kaya et al. (2023) clarified the mechanisms of action of salicylic acid in terms of eliminating the harmful effects of ROS [5].

Chen et al. (2016) treated wheat seeds with two doses of salicylic acid and examined the effects of the antioxidant in the detoxification system [248]. They found that the lower dose (0.25 Mmol) stimulated enzymes capable of detoxifying ROS, but the higher dose (2.5 Mmol) inhibited it. Pirasteh-Anosheh et al. (2018) investigated barley plants under salt stress [249]. The higher hydrogen peroxide level indicated the plants were under strong oxidative stress. After applying salicylic acid, the enzyme activities of peroxidase, catalase, superoxide dismutase, and glutathione reductase increased, and secondary oxidative damage was suppressed.

Ahanger et al. (2020) examined *Vigna angularis* plants under salt stress and found that secondary oxidative stress accelerated plant death [250]. However, salicylic acid treatments boosted the antioxidant system. The activities of enzymatic antioxidants, including superoxide dismutase, catalase, ascorbate peroxidase, dehydroascorbate reductase, and glutathione reductase, as well as the activities of non-enzymatic scavengers, eliminated the harmful effects of oxidative stress. Salicylic acid is a major regulator of oxidative stress. However, the underlying mechanisms remain largely unexplored. Saleem et al. (2021) aimed to review the molecular mechanisms of SA regulation of redox homeostasis [224].

Lukan and Coll (2022) further considered the role of salicylic acid signaling in ROS neutralization [251]. They argued that the regulation is a reciprocal and self-regulatory mechanism between the SA pathway and the ROS system. The reciprocal nature of the mechanism is essential for stopping the advance of pathogens and for the development of a successful immune response. Kohli et al. (2022) found that nitric oxide, itself a reactive free radical, combined with salicylic acid helps to neutralize ROS [252]. This nitric oxide-mediated immune response is achieved by the plant by upregulating salicylic acid pathway-related pathogen-related genes (NPR-1 and NPR-2).

Moustakas et al. (2022) demonstrated that salicylic acid offers protection to the PSII photochemical system by reducing the chlorophyll content in the cells and thus reducing the number of excitable chlorophyll molecules available in the system, thus limiting the possibility of singlet oxygen formation [253]. In addition, the efficiency of the oxygen emission complex (OEC) increases, resulting in a decrease in hydrogen peroxide levels. Thus, the effects of ROS are ultimately reduced. Myers et al. (2023) compared the effects of plant hormones on stress-induced signaling pathways and found that while salicylic acid enhances ROS signaling, jasmonic acid reduces it [254]. They also point out that abscisic acid and ethylene are also involved in the fine-tuning of these processes, so the combined effect of plant hormones is essential in modulating stress-induced signaling pathways.

#### 2.1.11. Salicylic Acid and Oxidative Stress Caused by Xenobiotics

Stress from a particular class of anthropogenic stressors, known as xenobiotics, also increases oxidative stress. In many cases, we accidentally cause damage to our cultivated plants by improper handling and applying pesticides. Let us just think of the application of chemicals under inappropriate weather conditions or the accidental damage caused by chemicals left in the tank. A good part of higher plants has a defense system that provides some protection against the phytotoxicity of xenobiotics. Some plants have a natural tolerance to substances that kill other plants. Some cultivated plants are able to break down toxic substances within the cell, transforming them into harmless compounds.

This can happen by means of oxidation or hydrolysis, which is then followed by further reactions, but unfortunately this method is not very effective because labile compounds are often formed, which are easily converted back into the initial phytotoxic substances.

The antioxidant system and the most important thiol compound of the cell, glutathione, play a crucial role in the detoxification of a large group of herbicides. After the formation of a conjugate with glutathione (with the help of the enzyme glutathione-S-transferase), the toxic compounds are transported to the vacuole. Several research groups have used salicylic acid treatments over the past decade to mitigate the damage caused by xenobiotics. Singh et al. (2017) studied the oxidative effects of the active ingredient glyphosate on tomato plants [204].

They found that it negatively affected shoot and root length and the fresh and dry weight of the plants and inhibited the activities of carotenoids, chlorophylls, protein synthesis, and nitrate reductase. Furthermore, they observed secondary oxidative stress effects, characterized by elevated hydrogen peroxide levels and ROS-induced membrane damage, which resulted in lipid peroxidation and electrolyte leakage. To counteract oxidative processes, salicylic acid treatments activated the function of certain antioxidant enzymes (superoxide dismutase and guaiacol peroxidase) and upregulated the key enzyme of the shikimic acid pathway, phenylalanine ammonia lyase, which is responsible, among other things, for the production of secondary protective compounds.

Spormann et al. (2019) also protected plants against the oxidative effects of glyphosate with salicylic acid in barley [255]. The xenobiotic caused the accumulation of hydrogen peroxide and superoxide anions, resulting in significant membrane damage. Additionally, we observed an increase in proline and non-protein thiols, as well as a decrease in the reduced ascorbate enzyme content. Salicylic acid had a moderately positive effect on plant homeostasis because it raised the levels of enzymes like catalase, ascorbate peroxidase, glutathione-S-transferase, and superoxide dismutase. However, the harmful effects of glyphosate still affected the plants.

Radwan et al. (2019) treated hazelnut plants with Basagran and then protected them with salicylic acid against the damaging effects [256]. That which does the work in the mixture is bentazone, a photosynthesis-inhibiting herbicide that goes after the PS-II photochemical system. The treatments triggered the activation of peroxidases, catalase, and ascorbate peroxidase, leading to the formation of a significant amount of phenolic compounds, which collectively mitigated the effects of oxidative stress. Guan et al. (2019) treated tobacco plants with sewage sludge contaminated with triclosan pesticide residue [257]. Triclosan is a potent, broad-spectrum antibacterial and antifungal disinfectant. The xenobiotic inhibits plant growth by limiting photosynthesis, reducing chlorophyll content, and inducing the overproduction of reactive oxygen species. Salicylic acid treatments modulated detoxification processes by increasing reduced glutathione (GSH) content, while inducing GR activity and GSH synthetase (GSHS) gene expression.

Liu et al. (2021) applied three pesticide active ingredients to cucumbers and then treated the plants with salicylic acid [258]. The three active ingredients were clothianidin (CLO), dinotefuran (DFN), and difenoconazole (DFZ). CLO is a second-generation nicotine derivative with insecticidal activity. DFN acts on the synaptic, nicotinergic acetylcholine receptors of the insect nervous system, while DFZ is a triazole fungicide that inhibits ergosterol biosynthesis in pathogenic cells. Some of the bad things they did were lower biomass, lower chlorophyll content, increase reactive oxygen species and proline buildup, increase lipid peroxidation, and cause changes in the activity of several antioxidant enzymes. However, salicylic acid treatments significantly reduced the half-life of the active ingredients and activated the plants’ degradative and detoxifying processes. Kumar et al. (2023) reviewed salicylic acid’s endogenous and exogenous effects [259]. They talked about the signaling pathways and genes activated by salicylic acid treatments. These include genes that deal with glutathione metabolism, glyoxylate and dicarboxylate, secondary metabolite synthesis, P450, antioxidant enzymes, and ABC transporter subfamilies. They also talked about how these genes help eliminate oxidative stress.

### 2.2. New Perspectives

#### 2.2.1. Effects of Salicylic Acid on Secondary Metabolite Production

Plants switch biochemical processes to alternative metabolic pathways in response to stress. These alternative pathways (e.g., the pentose phosphate pathway) are typically directly connected to secondary metabolic pathways such as the shikimic acid pathway, which produces secondary metabolites. These secondary metabolites are part of plants’ defense mechanisms, including numerous antioxidant and antimicrobial compounds. These pathways also produce various phenolic compounds.

In the past decade, several research groups have conducted studies to determine how salicylic acid treatments can alter the synthesis of secondary metabolites. Gorni et al. (2016) treated yarrow plants with salicylic acid to stimulate the production of secondary metabolites [260]. It was found that salicylic acid applied at concentrations of 0.5 and 1 Mmol significantly increased the production of some antioxidant oils in the plants, thereby increasing the nutritional and medicinal values of this species and improving the economics of its commercial use.

Su et al. (2018) investigated the possible positive effects of salicylic acid on increasing the amount of some secondary metabolites—baicalin and bicalein—in *Scutellaria baicalensis Georgi* plants [261]. They found that the application of salicylic acid was positively correlated with the synthesis of baicalin but negatively with that of bicalein. The phenylalanine ammonia-lyase and isochorismate synthase pathways are known to make salicylic acid in the body. The experiment also showed that adding salicylic acid from outside the cell positively affected the activity of the two biosynthetic pathways. Therefore, the exogenous application of salicylic acid to increase the intensity of endogenous salicylic acid production may be a promising perspective. Ali (2021) discussed the results so far on the effects of salicylic acid on the production of secondary metabolites, concluding that in addition to its stress-relieving effects, it can also provide an environmentally friendly, alternative solution for increasing the amount of internal parameters, including secondary metabolites [262].

#### 2.2.2. Salicylic Acid as Priming Effector

Some researchers have also tested the active ingredient salicylic acid for priming plants. Shatpathy et al. (2018) found that plants grown from seeds primed with SA had increased seedling weight, root and shoot length, and shortened germination time compared to untreated seeds [263]. Thus, it has a proven beneficial effect on the growth of healthy seeds as well. The same results were obtained by Mahmood-ur-Rehman et al. (2020) on primed carrot plants [264]. Galviz-Fajardo et al. (2020), on the other hand, found that seed priming with SA did not improve tomato seed germination and early seedling growth [265]. Ceritoğlu and Erman (2020) also measured that 0.2 mM salicylic acid priming is the threshold value for chickpea, and higher concentrations inhibit the germination process [266].

In addition to the uses of salicylic acid described so far, researchers have also noticed an important new mechanism of action. Salicylic acid can also be an effective tool for activating plant immunity. When applied in the right concentration and time, it can activate the plant immune system as an eustressor compound even before the onset of stress effects and induce protective responses in plants through signal transduction mechanisms. Szalai et al. (2016) primed corn seeds with salicylic acid and found that this had a beneficial effect on polyamine metabolism, which may be indirectly related to the stress tolerance of plants [267]. Mahesh et al. (2017) treated *Solanum melongena* seeds with SA and found that it activates PR proteins and their associated defense enzymes (β-1,3-glucanase and chitinase) upon exposure to an infectious agent [268].

Zanganeh et al. (2018) primed maize seeds with SA and observed that the pretreatment effectively influenced the role of methionine and arginine metabolism in plants under heavy metal stress conditions [269]. Zanganeh et al. (2019) seed-primed maize and then exposed the growing plants to lead stress [195]. Stressed but primed plants made more ascorbic acid, glutathione, and antioxidant enzymes. Pretreatment with SA also increased l-cysteine desulfhydrase’s activity and the amount of hydrogen sulfide that plants naturally produce. They found that hydrogen sulfide is a downstream signaling molecule that may be involved in SA-induced responses. Bortolin et al. (2020) also showed that Trifolium vesiculosum and Trifolium repens seedlings grown from primed seeds tolerated aluminum toxicity better than untreated specimens, as they showed increased APX and SOD activity [270].

Methenni et al. (2018) applied salicylic acid priming to olive plants against salt stress [271]. The priming process significantly reduced Na+ accumulation in the plants, alleviating the harmful effects of salt stress. Gharbi et al. (2018) primed plants with salicylic acid and compared the efficacy of the treatment with the results of plants exposed to salt stress and subsequently treated with salicylic acid [272]. They found that both treatments were effective but involved different biochemical pathways. Similarly, Ghafoor et al. (2020) applied priming treatments to wheat and then subjected the plants to salt stress [273]. They also found that primed plants were more resistant to salt stress, increasing their physiological parameters such as root length, shoot length, seedling weight, and vitality. Iqbal et al. (2021) primed maize seeds with SA and exposed them to salt stress [274]. They found that seed priming with 30 mg/L salicylic acid solution effectively increased uniform seed germination and the germination rate under stress conditions.

Islam et al. (2022) found that exogenously applied salicylic acid (SA) seed priming alleviated the adverse effects of salinity on plants in baby corn plants [275]. The application of 1 mM concentration gave the most promising results in terms of root dry matter, cob yield, leaf relative water content, and free proline content. Ahmad et al. (2022) primed pea plants and then exposed them to salt stress [276]. By modulating both enzymatic and non-enzymatic antioxidant systems, inducing the accumulation of soluble sugars and proline, regulating ion homeostasis, and enhancing the performance of Na^+^/H^+^ antiporters, priming mitigates damage even at higher salinity levels. Chickpea plants were primed by Kaur et al. (2022), and under salt stress, it was found that the treatments increased biomass, photosynthetic efficiency, slowed plant senescence, and reduced oxidative stress [13].

Ben Youssef et al. (2023) primed barley seeds with SA and exposed them to salt stress. They found that salinity negatively affected shoot and root growth, transpiration rate, chlorophyll concentration, stomatal conductance, and water content in plants from untreated seeds [277]. Primed seeds improved all of these traits in plants compared to untreated ones. Maqsood et al. (2023) primed wheat and exposed the emerging plants to salt stress [278]. They found that salicylic acid seed priming significantly improved fresh root mass, chlorophyll b content, POD activity, shoot Ca^2+^ content, and total yield.

Ayub et al. (2020) primed okra plants with salicylic acid and exposed them to drought stress [279]. They discovered that a concentration of 2 Mmol of the active ingredient most effectively reduced the adverse effects. Tayyab et al. (2020) primed maize with SA, and the pretreated plants successfully maintained their turgor under drought stress treatments [280]. The levels of catalase, peroxidase, and superoxide dismutase enzymes were significantly increased by priming. Alam et al. (2022) found that seed priming had a beneficial effect on cantaloupe quality parameters during water deficit stress. In particular, the membrane stability index, dry matter content, and yield were prominent [281]. Kulak et al. (2021) primed basil plants with SA and exposed them to water deficit stress [282]. Priming effectively increased plant physiological parameters and increased phenolic and flavonoid content. However, the treatment did not significantly affect the percentage of essential oil components in basil plants. Chakma et al. (2021) primed grape tomato seeds and applied them at a concentration of 100 mg/L, achieving a 33% yield increase during drought stress compared to the control plants [283].

#### 2.2.3. Salicylic Acid and Nanoparticles

Using nanoparticles, a new technique to improve crop production indicators, is a promising method. In addition to its many advantages, its stress-relieving effects are increasingly being reported [284]. Furthermore, we summarize the knowledge available about its application possibilities in combination with salicylic acid. Souri et al. (2017) prepared and tested salicylic acid nanoparticles on *Isatis cappadocica* plants under arsenic stress [285]. They found that the amount of arsenic accumulated in the roots and shoots of treated individuals of this plant species, which plays a vital role in soil bioremediation, increased. However, there was no significant change in the plant’s homeostasis. In conclusion, it can be stated that the plant can withstand even increased heavy metal loads after pretreatment with salicylic acid nanoparticles.

Mozafari et al. (2018) treated strawberries in vitro with a combination of iron oxide nanoparticles and salicylic acid and then exposed the plants to drought stress [286]. They found that the combined treatments promoted plant development and improved stress tolerance. Abdoli et al. (2020) applied iron oxide nanoparticles in combination with salicylic acid to ajowan plants under salt stress [287]. The treatment made the plants take in more K^+^, helped keep the K^+^/Na^+^ ratio stable, raised the levels of iron and salicylic acid in the plants, and increased the production of antioxidant enzymes (superoxide dismutase, catalase, peroxidase, and polyphenol oxidase) and osmolytes. This improved the homeostasis of stressed plants. Ghassemi-Golezani et al. (2021) also treated ajowan plants with a combination of iron oxide nanoparticles and salicylic acid under salt stress [288]. They found that the treatments increased the activities of hydrogen ATPase and hydrogen PPase enzymes, thereby reducing harmful Na^+^ uptake and maintaining stable ion balance.

Faizan et al. (2021) reported the beneficial effects of zinc oxide nanoparticles in combination with salicylic acid in rice plants under arsenic stress [289]. They found that combining nanoparticles and SA regulated the transcription of genes responsible for eliminating oxidative stress. In addition, they had a beneficial effect on maintaining ion balance and the uptake of both macro- and microelements. Al-Taey and Al-Musaw et al. (2022) applied nano-NPK fertilizer and salicylic acid to rocket plants under salt stress [290]. They found that the leaves’ content of glucosinolates, ascorbic acid, and glutathione increased, thus compensating for the adverse effects of salt stress.

Zhong et al. (2023) prepared salicylic acid (SA)-functionalized mesoporous silica nanocarriers and loaded them with salicylic acid [291]. They tested their effects on cucumber plants and successfully combated the damaging effects of salt stress with their help. In addition, the so-called nano-pesticide produced this way had a powerful insecticidal effect. It may be particularly intriguing to plan future experiments with this nano-pesticide. Yan et al. (2023) grafted salicylic acid onto a zein-based nano-pesticide carrier, which provides a promising new opportunity to reduce the attack of biotic stressors and the effects of salt and alkaline stress [89]. Aazami et al. (2023) treated grapes with a chitosan-based salicylic acid nanocomposite (CS-SA NCs) under normal conditions and during salt stress [292]. They found that the applied treatment has a biostimulant effect during the non-stressed period, while during salt stress, it activates the antioxidant defense system and increases the total soluble carbohydrate and protein content in the plants.

Yin et al. (2024) tested nanoparticles made from a combination of salicylic acid (SA) and six nanomaterials on cotton plants, including mesoporous silica nanospheres (MSNs), mesoporous Fe_3_O_4_ nanospheres (Fe_3_O_4_), carbon quantum dots (CQDs), polylactic acid-glycolic acid copolymer (PLlicb), and hydrophilic cation copolymer (HLDP) [293]. The HLDP–salicylic acid combination provided the most effective protection against biotic stressors. They mainly observed fungicidal effects, which may open the way for new applications.

#### 2.2.4. Salicylic Acid in Combined Treatments

After showing that salicylic acid has its effects, researchers thought it might be helpful to use the active ingredient and other substances that increase plant yield, relieve stress, or boost the immune system, to see if the combined effects would be even better for the plants. Sedaghat et al. (2017) used SA applied in combination with strigolactones in winter wheat against drought stress [294]. They found that the two compounds’ synergistic effects enhanced the plants’ drought tolerance. The mix strengthened the membrane structure by adding more antioxidants, reducing electrolyte leakage and malondialdehyde levels. The results of the experiment were also confirmed by experiments with the duplicate content by Sedaghat et al. (2020) [295].

Maghsoudi et al. (2018) combined salicylic acid with silicon (Si) under drought stress and found that treated wheat plants tolerated the adverse effects of stress better with the combined treatment than plants treated with one or the other agent alone [296]. Gene expression studies supported the differences between the treatments and found a significant difference in the increase in the activity of the Δ1-pyrroline-5-carboxylate synthetase (P5CS) gene. Their experiments showed that the P5CS gene is stress-induced and, together with some transcription factors, helps the plant defend itself against drought stress. Similarly, Khalequzzaman et al. (2024) combined SA with silicon on cotton plants under drought stress and also reported beneficial synergistic effects [297].

Kohli et al. (2018) combined salicylic acid with a brassinosteroid compound called 24-epibrassinolide (EBL) and studied the effects of SA and EBL on heavy metal stress in *Brassica juncea* plants [298]. They found that the combined effect of SA and EBL was most effective on plants suffering from lead stress. They showed that the treatment activated the entire antioxidant system, including non-enzymatic antioxidants. They pointed out that EBL increased the expression of catalase, peroxidase, glutathione reductase, glutathione synthetase, and dehydroascorbate reductase genes and enhanced other beneficial effects of SA.

Hernández-Ruiz and Arnao (2018) summarized the results of studies on the combined effects of salicylic acid and melatonin [299]. Zulfiqar et al. (2023) investigated the combined effects of salicylic acid and melatonin on ornamental sword lilies under arsenic stress [300]. They found that the combined treatment considerably lessened the harmful effects of arsenic by raising the levels of protein and proline and the activity of enzyme-based antioxidants. Rafique et al. (2024) also associated SA with melatonin in rapeseed [301], and Kaya et al. (2023) made similar associations with tomato during drought stress [5]. They obtained similar experiences to those of the previous authors, thus supporting the close relationship of signaling pathways in forming defense responses.

Bijanzadeh et al. (2019) combined salicylic acid with humic acids (HAs) and tested how it affects the physiological processes of maize plants during drought stress [302]. They found that pigment content, relative water content, root and shoot length, and K^+^ accumulation were higher when the two active ingredients were combined. HA supports the absorption and incorporation of other active ingredients, thus enhancing the performance of the main compound.

A synergistic effect was observed by Faraz et al. (2020) when the combined application of salicylic acid and citric acid (CA) was applied to *Brassica juncea* plants under heavy metal stress [303]. The combined treatment increased the width of the stomatal pores like no other treatment did. This changes the internal CO_2_ concentration and carbonic anhydrase activity, improving photosynthesis in plants. Emamverdian et al. (2020) investigated the combined effects of gibberellic acid (GA) and SA and found that GA has a beneficial effect on the internal SA level of plants, thereby exerting a stimulatory effect on the development of plant defense responses [304].

Farhangi-Abriz and Ghassemi-Golezani (2018) investigated the combined application of SA and jasmonic acid (JA) in salt stress [61]. Since the two compounds have antagonistic effects on plant defense responses [305], the researchers focused on different target areas. They found that SA increased calcium and potassium uptake, glycine betaine content, and cell levels of soluble sugars, proteins, and antioxidant enzymes. SA also increased leaf water content, membrane stability index, chlorophyll content, and chlorophyll stability index, but decreased proline content. In contrast, JA decreased sodium uptake, but similarly to SA, glycine betaine and soluble protein content, antioxidant enzyme activity, membrane stability index, and leaf water content increased. However, the soluble sugar content, potassium and calcium ion content, chlorophyll content, and chlorophyll stability index did not change. The antagonistic effect of internal signal transduction pathways was not detectable in the effect of externally applied plant conditioning treatments, and there were even jointly modulated biosynthetic pathways.

Sofy et al. (2020) applied salicylic acid in combination with proline (Pro) and/or jasmonic acid (JA) to maize against heavy metal stress [306]. They found that the combination of JA, SA, and Pro enhanced plant growth, induced pigment biosynthesis, and reduced heavy metal uptake. All the treatments and combinations also increased the production of glutathione, catalase, superoxide dismutase, peroxidase, endogenous Pro, and total soluble sugar. As a result, we reduced the harmful ROS effects without causing any damage to the membranes. Since the membranes remained intact, electrolyte leakage and malondialdehyde concentrations were reduced.

Prakash et al. (2021) summarized the published knowledge on the combined effects of SA and nitric oxide (NO) in their review article and concluded that NO, when applied externally in small amounts, activates the action mechanisms modulated by SA as a signaling molecule, while in large amounts, it directly helps to eliminate plant stress [307]. Rai et al. (2021) also discussed their essential role in responses to biotic stress in their summary work [308]. They concluded that SA and NO may be one of the immune response reactions that are unavoidable by biotrophic pathogens, due to the mutually reinforcing nature of their effects.

Rasheed et al. (2022) investigated the combined effects of SA and sulfur (S) as stress-relieving compounds in salt stress. The individually effective active ingredients synergistically affect plant stress relief [309]. Munsif et al. (2022), similar to the previous study, discovered the synergism of SA and potassium (K) during drought stress [310]. The combined application increased plants’ photosynthetically active pigment forms and leaf water content, and the water balance of plants became more stable.

Mabudi Bilasvar et al. (2022) investigated the combined effects of a polyamine compound, putrescine, and SA in rapeseed exposed to drought stress [311]. The combined treatment raised the amount of soluble sugars and the osmoprotectant glycine betaine in rapeseed while lowering the amount of proline in the leaves. In addition, the oil content increased because the treatments prolonged the filling duration of the seeds, despite the stress. However, the fatty acid composition improved only when SA was applied alone.

#### 2.2.5. Salicylic Acid and Mycorrhiza Connections

In the previous chapter, we discussed the possible combinations of salicylic acid with other compounds and their benefits for plants. However, it is not only possible to combine SA with other chemicals. In this chapter, we will explore the application possibilities of salicylic acid in combination with living biological organisms and the experiences gained with them. Benjamin et al. (2022) reviewed the symbioses of plants with endophytes, arbuscular mycorrhizal fungi, and nitrogen-fixing rhizobia [312]. They concluded that the primary supporting role of microorganisms is in alleviating stress effects.

Islam et al. (2016) treated chromium-stressed maize plants with a combination of SA and chromium-resistant plant growth-promoting bacteria (PGPB) [313]. They found that the treatments reduced chromium toxicity and oxidative stress and improved plant function, leading to better yield parameters. They also found that the combined treatment was more effective than SA or PGPB treatment alone. A combination of plant growth-promoting rhizobacteria (PGPR) and salicylic acid was tested by Khan et al. (2018) in sunflowers under drought stress [314]. They also evaluated their role in phytoremediation. They found that the treatment increased the accumulation of copper, zinc, and cobalt but reduced iron accumulation in plants. All these effects were particularly significant even in water-stressed environments.

Garg and Bharti (2018) treated chickpea plants under salt stress by seed priming with a combination of SA and an arbuscular mycorrhiza (*Rhizoglomus intraradices*) [315]. The symbiosis was effective against stress, especially by enhancing sugar export to the fungi, which benefited fungal growth. The combined priming increased root biomass, root-to-shoot ratio, and nutrient uptake of the stressed plants, offering a promising perspective for the future. Azmat et al. (2020) treated drought-stressed wheat with SA and rhizobacteria-formulated biofertilizer (RBF) [316]. They found that the combined application increased the viability and size of the bacterial population and helped restore vital functions under stress. Both chlorophyll content and carotenoid biosynthesis increased. Additionally, we observed the activation of the antioxidant enzyme system. The combined application of SA and BF resulted in better drought tolerance than the application of SA or RBF alone.

Nigam et al. (2022) treated spinach and soybean plants exposed to high salt concentrations with a combination of Stenotrophomonas sp. and salicylic acid [317]. The enzyme ascorbate peroxidase levels went up and new proteins showed up. These were signs that the combined treatment was working well. Ali et al. (2023) studied maize plants under salt stress [87]. The plants’ seeds were inoculated with a salt-tolerant strain of Pseudomonas aeruginosa, and salicylic acid was subsequently applied as foliar fertilizer. The combined application significantly alleviated the adverse effects of salt stress. Together, the rhizobacterium and salicylic acid increased the activities of ascorbate peroxidase, catalase, and superoxide dismutase in the leaves. They also fixed the Na^+^/K^+^ ratios. These results are promising for future perspectives regarding the exogenous applications of PGPBs and salicylic acid together.

Boamah et al. (2023) summarized the beneficial effects of *Trichoderma* fungi during biotic (*Fusarium* infection) and abiotic (salt stress) stress [318]. They say that the mixture of fungi increases the activity of genes involved in the body’s natural salicylic acid biochemical signaling pathway, which helps lower the effects of stress. We believe further research perspectives may lie in using a combination of *Trichoderma* and exogenous salicylic acid.

## 3. Conclusions

Extreme weather events resulting from global climate change and adverse anthropogenic impacts greatly affect today’s agricultural productivity. Researchers must find compounds that can improve plant health and value traits in a way that is safe for the environment and works well. These compounds should be able to increase yields, general reproduction, immune system activity, and plant conditioning. These challenges encourage researchers to closely examine key compounds endogenously present in plants that can promote plant defense mechanisms against environmental challenges.

Plants rely heavily on salicylic acid-based signaling and its interactions with other defense pathways. In addition to influencing plant physiological and biochemical functions in a dose-dependent manner, SA is involved in the induction of defense-related genes and stress resistance [319]. External salicylic acid treatments have a positive effect both before stress effects occur, when applied as a primer, and after they have occurred, eliminating their harmful effects. When combined with other active ingredients, they have many possible uses, allowing for a wide range of applications in agriculture.

Since it is highly effective even in low concentrations, its range of uses may expand with the development of drone application technology. In addition to all these positive properties, an important aspect is that—since it is also produced endogenously in the plant—it is a natural, broad-spectrum active ingredient that can be effectively integrated into environmentally conscious production systems, thus helping to increase the efficiency of healthy food production and at the same time reduce our ecological footprint.

## Figures and Tables

**Table 1:** The most important literature references from the last decade regarding using salicylic acid under salt stress conditions.

Plant Species	Target Areas of Beneficial Effect	References
**Barley**	Grain number	[64]
	Proline content, indole-acetic acid content	[65]
**Barley and Wheat**	Higher grain yield	[64]
**Bean**	Concentrations of total chlorophylls, total carotenoids, total soluble sugars, free proline and ascorbic acid, contents of N, P, K, and Ca, and ratios of K/Na and Ca/Na	[66]
	Improve shoot length, number, and area of leaves per plant, plant dry weight	[67]
	Free proline, soluble sugars, chlorophyll fluorescence, relative water content, and membrane stability index	
	Maintaining an enhanced pool of reducing agents	[68]
	Leaf pigment contents, ascorbic acid, glutathione, proline, K, and antioxidant enzyme contents	[69]
**Canola**	Shoot fresh weight, root dry weight, chlorophyll content, and antioxidant enzyme activities	[70]
**Chinese Cabbage**	Photosynthetic performance, productivity, and photosynthetic pigment content	[71]
**Cotton**	Increasing K^+^ and Mg^2+^ absorption and osmotic regulators accumulation	[72]
	Photosynthetic parameters, yield parameters	[69]
	Decrease in the accumulation of hydrogen peroxide, and the elevated levels of electrolyte leakage and malondialdehyde	[73]
**Cowpeas**	Total free amino acids and shikimic acid	[74]
**Cucumber**	Regulate endogenous salicylic acid levels	[75]
**Damask Rose**	Activity of enzymatic and non-enzymatic defense systems	[76]
***Dianthus superbus***	Stoma and chloroplast development	[77]
	Leaf biomass, soluble protein, sugar content, expression of MYB and P5CS genes	
**Eggplant**	Activate pigments	[78]
***Egletes viscosa***	Increase in the content of all organic compounds, decrease in H_2_O_2_ overproduction	[79]
**Ethiopian Mustard**	Proline content, antioxidant enzyme activities	[80]
**Fenugreek Plants**	Decrease of calcium, potassium, and phosphorus concentrations	[81]
**Feverfew**	Increase in the essential oil, sugar, and antioxidant contents	[82]
***Limonium bicolor***	High levels of gibberellic acid (GA) and high levels of amylase and α-amylase activity	[83]
**Maize**	Proline concentration, amino acid accumulation	[84]
	Root and shoot growth, increased CO_2_ concentration	[85]
	Regulation of phytochromes and various organic and inorganic osmolytes	[86]
	Leaf osmolyte and sugar contents	[87]
	Activate the ascorbate–glutathione cycle	[88]
**Melon**	Activate MAPK cascade, plant hormone signal transduction, lipid metabolism, biosynthesis of secondary metabolites (phenylpropanoids and flavonoids)	[89]
***Mentha Pulegium***	Relative water content	[90]
**Mungbean**	Increasing photosystem II activity, decreasing fluorescence	[91]
	Photosynthetic efficiency	[92]
**Mustard**	Increase in ATP sulfurylase and serine acetyl transferase activity	[93]
	Neutralize the NaCl stress-induced suppression	[94]
***Nitraria tangutorum***	Antioxidant capacity, ascorbate–glutathione cycle	[95]
**Okra**	Antioxidant enzyme activity	[96]
	Improving plant properties in hydroponic system	[97]
**Olive**	Chlorophyll index	[98]
**Pea**	Superoxide dismutase, catalase, guaiacol peroxidase and ascorbate peroxidase activity	[99]
**Pearl Millet and Wheat**	Increase of the dissipation flux in the photosynthesis	[100]
**Pennyroyal**	Content and antioxidant activity of essential oils, nutrient uptake, photosynthetic activity, plant growth	[101]
**Pepper**	Upregulate the activities of ascorbate–glutathione cycle	[102]
**Pomegranate**	Chlorophyll, total phenolic, carbohydrate, and proline content	[103]
**Radish**	Accumulation of compatible solutes, antioxidant activities	[104]
**Rice**	Expression of SOS1 and NHX1 genes	[105]
	MAPK-1, transcription factor WRKY53, Bax Inhibitor-1, and nine Autophagy Related Genes were upregulated	[106]
	Regulation of the expression of osdreb2a and ossapk8 genes	[107]
**Rosemary**	Total phenolic, chlorophyll, carbohydrates, and proline contents of leaves	[108]
**Safflower**	Relative water content, leaf area index, and chlorophyll content index	[109]
	Content of total phenolics, total flavonoids, total flavonols, anthocyanins, and antioxidant activity	[110]
	Increasing glycine betaine and anthocyanin content	[111]
	Chlorophyll content, antioxidant capacity	[112]
**Sage**	Appearance of the new majority compound thujanone	[113]
**Sorghum**	Osmolyte concentration, rates of gaseous exchange attributes, and antioxidant enzymatic activity	[114]
**Soursop**	Stomatal conductance, CO_2_ assimilation rate, transpiration, and carboxylation efficiency	[115]
**Soybean**	Leaf chlorophyll content index, anthocyanins content, leaf area, water use efficiency, seed filling duration, assimilate mobilization efficiency, and seed mass	[116]
	Oil content per soybean seed, seed yield	
**Strawberry**	Fresh and dry weight of shoots and roots, activity of ascorbate peroxidase, peroxidase, and superoxide dismutase enzymes	[117]
	Antioxidant enzyme activities, ascorbic acid level	[118]
**Sunflower**	Uptake of K^+^ ion	[119]
**Tomato**	Upregulation of ABA biosynthesis genes, zeaxanthin epoxidase, 9-cis-epoxycarotenoid dioxygenase, and aldehyde oxidases	[120]
	Increase in the ascorbate-peroxidase and glutathione reductase activity	[121]
	Leaf concentration of sodium, proline, and soluble sugars	[122]
**Wheat**	Net photosynthetic rate, transpiration rate, stomatal conductance, maximum quantum efficiency of PSII photochemistry, actual photochemical efficiency of PSII, electron transport rate, photochemical quenching coefficient, effective quantum yield of PSII photochemistry	[123]
	Accelerating the metabolic flow, promoting nitrogen metabolism	
	ATP content and H^+^-pump activity of the roots	[124]
	Upregulation of the glyoxalase system and ascorbate–glutathione cycle	

**Table 2:** The most important literature references of the last decade regarding the use of salicylic acid under drought stress conditions.

Plant Species	Target Areas of Beneficial Effect	References
***Ammi visnaga***	Two major secondary metabolite γ-pyrones, the khellin and visnagin content	[142]
***Aristotelia chilensis***	Phenol content, antioxidant capacity	[143]
**Barley**	Stem length, plant dry weights, chlorophyll concentration, relative water content, activity of antioxidant enzymes, and grain yield	[144]
**Chinese cabbage**	Superoxide dismutase, catalase, guaiacol peroxidase, and ascorbate peroxidase content, upregulation of pyrroline-5-carboxylate synthase genes (P5CSA and P5CSB)	[137]
**Creeping bentgrass**	Accumulation of amino acids (proline, serine, threonine, and alanine) and carbohydrates (glucose, mannose, fructose, and cellobiose)	[145]
**Grape**	Carotenoids content, CAT, APX, and GPX enzymes activities	[146]
***Impatiens walleriana***	Expression of the gene Δ1-pyrroline-5-carboxylate synthetase (P5CS) and Δ1-pyrroline-5-carboxylate reductase gene (P5CR)	[147]
**Lemon verbena**	Improving physiological parameters and essential oil content	[148]
**Maize**	Antioxidant enzymes activity, proline and soluble sugar content	[149]
***Mentha pulegium***	Relative water content, proline accumulation, antioxidant enzymes activities (SOD, POX, and PPO)	[90]
**Mustard**	Increasing the proline production through the increase in γ-glutamyl kinase (GK) and decrease in proline oxidase (PROX) activity	[93]
	Water potential, osmotic potential, water use efficiency, photosynthetic nitrogen use efficiency	[150]
**Onion**	Membrane stability index, relative water content	[151]
**Pearl millet and wheat**	Plant height and grain yield	[100]
**Pumpkin**	Carbohydrate and fatty acid content	[152]
**Purslane**	Photosynthetic pigments, gas exchanges, compatible solutes and secondary metabolites	[153]
**Radish**	Shoot mass, storage root mass, parameters of gas exchange	[154]
**Rapeseed**	Leaf ultra-structures	[87]
**Rice**	Photosynthetic pigments, proline content	[128]
	Plant growth, dry weight, metabolism or metabolic activities, the nutritional status	[87]
**Rosemary**	Essential oil content and quality	[155]
***Ruta graveolens***	Essential oil content and composition	[156]
**Safflower**	Non-enzymatic defense system (scavengers)	[157]
	Rate of photosynthesis, anthocyanin content, phenylalanine ammonia lyase activity	[158]
**Sesame**	Growth and various physiological processes	[159]
	Net photosynthetic rate, stomatal conductance, leaf area index, chlorophyll a, b and total chlorophyll contents, maximum quantum efficiency of PSII, and plant dry matter and seed yield	[160]
	Osmoprotectant contents, antioxidant defense system, mineral nutrients in plant organs, photosynthesis	[161]
**Shallots**	Leaf relative water content, membrane stability index, chlorophyll content, onion yield	[162]
**Squash plant**	Chlorophyll fluorescence, osmoprotectants	[163]
**Strawberry**	Total leaf area, leaf and shoot dry matter, catalase and peroxidase activity	[164]
**Sunflower**	Total chlorophyll and carotenoid content	[165]
**Sweet basil**	Growth parameters, photosynthetic pigments, and relative water content	[166]
	Photosynthesis, antioxidant defense system	[167]
**Thyme**	P-cymene oil content, rosmarinic acid content, total polyphenolic content	[168]
	Thymol, carvacrol, linalool, p-cymene, and γ-terpinene content	[169]
**Tomato**	Antioxidant enzyme activities, net photosynthetic rate and water use efficiency	[170]
***Vicia faba***	Maintaining an enhanced pool of reducing agents	[68]
**Wheat**	Membrane stability, total flavonoids and total phenol contents, CAT and SOD activities	[171]
	Plant height, spike length, number of grains per spike, 1000 grain weight, chlorophyll content, relative water content	[172]
	Proline and soluble sugar content	[173]
	Carbon metabolism and signal transduction, energy production and protection in Lok1	[174]
	Accumulation of soluble sugars, potassium, magnesium, and calcium	[175]
	Enzymatic and non-enzymatic antioxidant system	[176]

**Table 3:** The most important literature references from the last decade regarding the use of salicylic acid under heavy metal stress conditions.

Plant Species	Heavy Metal	Target Areas of Beneficial Effect	References
***Arabidopsis thaliana***	Cadmium	Upregulation of electron transport-related and amylase-encoding genes	[189]
**Bean**	Cadmium	Superoxide dismutase, catalase, ascorbate peroxidase, and glutathione reductase	[190]
	Nickel and lead	Carbonic anhydrase, nitrate reductase, catalase, peroxidase, superoxide dismutase, photosynthetic pigment, carbohydrate contents	[191]
**Flax**	Cadmium	Levels of monogalactosyldiacylglycerol, phosphatidylglycerol, phosphatidic acid, and polyunsaturated fatty acids	[192]
**Lemon balm**	Mercury	Upregulation of chlorophyll synthase and phenylalanine ammonia-lyase genes as key components of chlorophyll and phenylpropanoid pathways	[193]
**Maize**	Copper	Seed metabolic activity, endogenous SA level, and carotenoid level	[194]
	Lead	Protein and glutathione contents, nitrate reductase activity	[195]
	Arsenic	Ascorbate–glutathione cycle, glyoxalase system	[196]
	Chromium	Accumulations of osmolytes, antioxidants, and endogenous polyamines	[197]
**Melon**	Cadmium	Chlorophyll content, photosynthetic activity, superoxide dismutase, guaiacol peroxidase, catalase, and ascorbate peroxidase activities, content of soluble protein and free proline	[198]
**Mustard**	Lead	Enzymatic and non-enzymatic antioxidant system	[199]
**Peanut**	Cadmium	Growth, chlorophyll content, photosynthesis, and mineral nutrition	[200]
**Pepper**	Lead	Growth parameters, biomass, leaf water status, and asa-GSH cycle-related enzyme activities	[201]
**Potato**	Cadmium	Relative water content, chlorophyll, proline, and endogenous SA contents	[202]
**Rapeseed**	Arsenic	Phytochelatin biosynthesis, S-assimilatory pathway, carbohydrate metabolism, rubisco, γ-glutamyl kinase, and proline oxidase enzyme activities	[203]
**Rice**	Arsenic	Endogenous levels of NO, nitrate reductase, and SA	[204]
	Cadmium	Photochemical activity of both photosystems, electron flow, energy distribution between pigment–protein complexes, kinetic parameters of oxygen evolution reactions	[205]
		Restricting Cd uptake and accumulation	[206]
**Ryegrass**	Cadmium	Uptake and translocation of mineral elements	[207]
**Tomato**	Cadmium	Catalase activity	[208]
**Wheat**	Arsenic	Malondialdehyde content	[209]

## Data Availability

All data are available within the manuscript.
